# Diagnostic accuracy of repeat placental growth factor measurements in women with suspected preeclampsia: A case series study

**DOI:** 10.1111/aogs.13818

**Published:** 2020-02-29

**Authors:** Kate E. Duhig, Louise M. Webster, Andrew Sharp, Carolyn Gill, Paul T. Seed, Andrew H. Shennan, Jenny E. Myers, Lucy C. Chappell

**Affiliations:** ^1^ Department of Women and Children’s Health School of Life Course Sciences King’s College London London UK; ^2^ Department of Women and Children’s Health University of Liverpool Liverpool UK; ^3^ Division of Developmental Biology and Medicine University of Manchester Manchester UK

**Keywords:** Diagnostic test, placental growth factor, preeclampsia

## Abstract

**Introduction:**

Preeclampsia affects about 3% of singleton pregnancies and is characterized by placental dysfunction. It is associated with significant maternal and perinatal morbidity and mortality. The diagnosis of preeclampsia remains a challenge, and the clinical course can develop for weeks before a diagnosis is confirmed. National guidelines have approved placental growth factor (PlGF) testing to rule out suspected preeclampsia, but the utility of repeated PlGF measurement is unknown. The aim of this case series analysis was to evaluate the test performance of repeated PlGF sampling in women presenting with suspected preeclampsia, and to describe relevant clinical outcomes.

**Material and methods:**

Women who presented to maternity services with suspected preeclampsia between 20^+0^ and 36^+6^ weeks’ gestation who underwent repeat PlGF sampling with a minimum test interval of 7 days were assessed. The outcomes were delivery for preeclampsia within 14 days of sampling, the proportion changing PlGF categories, and time to delivery.

**Results:**

In total, 289 women with suspected preeclampsia undergoing repeat PlGF sampling were included. PlGF <100 pg/mL had a high sensitivity (87.5%, 95% confidence interval [CI] 67.6%‐97.3%) and a negative predictive value (97.7%, 95% CI 93.5%‐99.5%) at the initial test (receiver operating characteristic [ROC] area 0.79, 95% CI 0.68‐0.89). Similar test performance was seen for PlGF <100 pg/mL when undertaken as a repeat test (sensitivity 90.7%, 95% CI 85.2%‐95.9%, negative predictive value 92.2%, 95% CI 85.3‐96.6%). Overall, 25.6% of women changed PlGF category between the first and second PlGF tests. For each PlGF category, determination of time to delivery was similar for first and second tests.

**Conclusions:**

Repeat PlGF measurement demonstrates high negative predictive value for determining preeclampsia requiring delivery in 14 days. Repeat testing may be clinically useful to risk stratify women with ongoing symptoms of disease. Confirmation of the impact of these findings is required in further studies.

**Abbreviations**
CI: confidence interval
IQR: interquartile range
PlGF: placental growth factor
sFlt‐1: soluble fms‐like tyrosine kinase 1
ROC: receiver operating characteristic

**Key message**

Repeat placental growth factor measurements demonstrate high negative predictive value for preeclampsia, and can risk stratify women with ongoing symptoms of disease.

## 1 INTRODUCTION

Preeclampsia affects 2.8% of singleton pregnancies and is characterized by placental dysfunction.^1^ It is associated with significant maternal and perinatal morbidity and mortality.^2^ One in 20 stillbirths without a congenital anomaly are attributed to, or associated with preeclampsia^3^ and it is a leading cause of iatrogenic preterm birth.^4^

The prediction and diagnosis of preeclampsia is an important component of antenatal maternity care; however, the diagnosis of preeclampsia remains a clinical challenge. Presentation is widely variable, and women with preeclampsia may be asymptomatic, even in the presence of severe disease. The clinical disease course can progress for weeks before a diagnosis is confirmed. The diagnostic criteria for preeclampsia, as defined by the International Society for the Study of Hypertension in Pregnancy, relies on the assessment of new‐onset hypertension and one or more features of multi‐organ disease.^5^ These signs and symptoms may be subject to substantial observer error^6^ and are frequently associated with poor test accuracy.^5^, ^7^, ^8^

The clinical uncertainty associated with diagnosing preeclampsia leads to the extensive use of laboratory investigations, and is frequently associated with admissions to hospital for inpatient monitoring. Advances in our understanding of the pathophysiology of preeclampsia have highlighted a role for placentally derived angiogenic factors as relevant disease biomarkers.^9^ Placental growth factor (PlGF) is a member of the vascular endothelial growth factor (VEGF) family, that in pregnancy is expressed predominantly in placental tissue, and correlates well with placental function. In normal pregnancies, PlGF is synthesized in abundance in the syncytiotrophoblast. Circulating PlGF concentrations rise throughout pregnancy until 30 weeks’ gestation, thereafter declining towards term. It is thought that placental hypoxia, oxidative stress, the release of pro‐inflammatory cytokines and reduced utero‐placental blood contributing to endothelial activation in preeclampsia causes a release of antiangiogenic factors and a subsequent reduction in maternal serum PlGF.^10^ A diagnostic accuracy study published in 2013 demonstrated that in women presenting with suspected preeclampsia, low circulating maternal PlGF concentrations (<100 pg/mL) had high sensitivity (96%; 95% CI 89‐99%) and negative predictive value (98%; 95% CI 93%‐99.5%) for diagnosing preeclampsia requiring delivery within 14 days.^11^ In the PARROT cluster randomized controlled trial, single PlGF measurements were revealed to clinicians alongside a clinical management algorithm to be used as a diagnostic adjunct in suspected preeclampsia. When compared with standard practice, there was a reduction seen in the median time to diagnosis of preeclampsia from 4.1 to 1.9 days (time ratio 0.36, 95% CI 0.15‐0.87) with a concurrent reduction in severe maternal adverse outcomes (5.4‐3.8%, adjusted odds ratio 0.32, 95% CI 0.11‐0.96).^12^

Of women who tested “normal” for PlGF (>100 pg/mL) in the PARROT trial, 10.9% went on to receive a diagnosis of preeclampsia in the pregnancy. In 2016, there was one small study of 100 women published which looked at repeated PlGF measurements in women presenting with suspected preeclampsia. This gave only limited descriptive outcomes of the participants, and no test performance characteristics.^13^ In 2019, Zeisler and colleagues published a post hoc analysis of participants in the PROGNOSIS study, in which repeated soluble fms‐like tyrosine kinase 1 (sFlt‐1)/PlGF measurements were undertaken on a weekly basis after initial presentation. This showed that there was a significantly larger median increase in sFlt‐1/PlGF ratio in women who subsequently developed preeclampsia or adverse fetal outcomes as compared with those who did not (mean difference 21.22 vs 1.40, *P* < .001). These repeated measurements were taken at fixed timepoints in all participants, not specifically in women with an ongoing suspicion of preeclampsia. The authors did not report test performance statistics for the repeat tests, which would enable confirmation that PlGF‐based tests retain similar test performance when used sequentially.^14^

We hypothesize that repeat PlGF sampling has high diagnostic accuracy in predicting the need for delivery for preeclampsia within 14 days. Repeat PlGF testing provides clinically important information in women in whom a diagnosis of preeclampsia remains uncertain following an initial test.

The aim of this analysis was to assess repeat sampling in a case series of women with suspected preeclampsia, to determine test performance of the first and second test for predicting preeclampsia requiring delivery within 14 days (as a marker of clinical disease severity) and to describe relevant clinical parameters of patients by the repeated test results.

## 2 MATERIAL AND METHODS

The data for this case series were obtained from pregnant women undergoing repeat PlGF testing from the PELICAN,^11^ MAPPLE (UK centers only),^15^ MAVIS/Manchester Placenta Clinic,^16^ PEACHES^17^ and PARROT^12^ studies, collating samples and data from 11 UK maternity units. Participants with repeat samples were included from these cohorts, which spanned the period from January 2011 to October 2018.

Women were included in this case series if they had two or more blood samples taken for PlGF in the index pregnancy for the indication of suspected preeclampsia. Women with a singleton pregnancy had the first blood sample taken at clinical presentation with suspected preeclampsia which included one or more of the following signs or symptoms: hypertension, dipstick proteinuria, headache with visual disturbances, epigastric pain, and fetal growth restriction between 20^+0^ and 36^+6^ weeks’ gestation. Women recruited with fetal growth restriction needed to have further signs or symptoms that led to clinical suspicion of preeclampsia. PlGF samples were repeated if women presented to antenatal services with an ongoing clinical suspicion of disease, and all second PlGF samples were taken 7 days or more from the first test. Women were excluded from this analysis if the repeat PlGF measurement was taken less than 7 days from the first test. Management decisions were left to the treating clinician’s discretion.

Blood samples were taken from women into bottles containing ethylenediamine tetra‐acetic acid, and tested for PlGF concentration following centrifugation on a Triage (Alere, now Quidel Cardiovascular Inc.) instrument, according to the manufacturer’s instructions. The sample results were in some cases revealed to the clinical term (in 131 women) and masked in others (in 158 women). Anonymized clinical data were collected in all cases in real time. Pregnancy outcome data were captured from the electronic patient records after birth. Preeclampsia was defined by the International Society for the Study of Hypertension in Pregnancy 2014 statement.^18^ All data were checked for completeness and adjudicated by the lead investigators of each study. Missing data from electronic records were retrieved from case notes where possible.

### 2.1 Statistical analyses

Women were classified according to the gestation of the test (<35 and 35‐36^+6^ weeks’ gestation).

Women were categorized by their measured PlGF concentration into the following predetermined groups:

1. PlGF ≥100 pg/mL—determined as “normal”
2. PlGF 12‐99 pg/mL, equivalent to <5th centile for gestation and determined as “low”
3. PlGF <12 pg/mL, the lowest limit of detection for the assay and determined as “very low”

These categorical groups were used based on the evidence that in those presenting <35 weeks’ gestation, “low” PlGF has a high diagnostic accuracy (0.96; 95% CI 0.89‐0.99) and negative predictive value (0.98; 95% CI 0.93‐0.995) of determining preeclampsia requiring delivery in 14 days in a prospective observational cohort study,^11^ and “very low” PlGF is the lowest limit of detection of the assay. We have previously reported that a PlGF threshold of <100 pg/mL predicted preeclampsia requiring delivery within 14 days or before 37 weeks’ gestation (whichever was sooner) with sensitivity and negative predictive values similar to diagnostic accuracy estimates obtained by using a <5th centile cut‐off. The study reflects national guidance, which recommends that PlGF is implemented as a rule‐out test up to 34^+ 6^ weeks’ gestation.^11^

For further sub‐classification of normal and low values, visual inspection of the data was undertaken and clinically applicable thresholds explored.

A positive test was PlGF <100 pg/mL. Test performance for the first test, and the repeat test for the primary endpoint of preeclampsia requiring delivery within 14 days (or before 37 weeks’ gestation in those recruited between 35^+0^ and 36^+6^) was calculated using sensitivity, specificity, positive and negative predictive values, positive and negative likelihood ratios, and receiver operating characteristic (ROC) areas, all with 95% confidence intervals. Test performance for the repeat test following an initial normal (PlGF >100 pmg/mL) test was also calculated. Median and interquartile ranges for time from second PlGF test to delivery were calculated, stratified by the result of the first test. Birthweights are presented as birthweight centiles.^19^ An additional sensitivity analysis of the test performance was performed on samples where the results were not revealed to clinicians in order to avoid treatment paradox. All statistical analyses were undertaken using STATA version 14.2 (StataCorp, College Station, TX).

### 2.2 Ethical approval

Ethical permission was obtained for research studies: PELICAN (East London Research Ethics Committee (ref. 10/H0701/117), PEACHES (ref. 11/LO/1776), and PARROT (ref. 15/LO/2058), but was not required for MAPPLE^15^ or MAVIS,^16^ as these were service evaluations using anonymized data.

## 3 RESULTS

In this case series, between January 2011 and October 2018, 289 women underwent repeated PlGF measurement. Of these, 149 were from the PELICAN study, 13 from the MAPPLE study, 58 from the MAVIS study, 44 from the PARROT trial and 25 from the PEACHES cohort study. Participant characteristics are given in Table 1.

**Table 1:** Maternal characteristics and pregnancy outcome

	All women n = 289	Revealed PlGF n = 209	Masked PlGF n = 80
Maternal age, y, mean (SD)	32.1 (6.0)	32.2 (6.2)	31.7 (5.5)
Body mass index, kg/m^2^, mean (SD)	28.8 (6.4)	28.9 (6.1)	28.7 (7.2)
Ethnicity			
White	184 (63.7%)	133 (63.6%)	51 (63.7%)
Black	66 (22.8%)	49 (23.4%)	17 (21.3%)
Asian	22 (7.6%)	14 (6.7%)	8 (10.0%)
Mixed	14 (4.8%)	11 (5.3%)	3 (3.8%)
Other (including Chinese)	3 (1.0%)	2 (1.0%)	1 (1.3%)
Parity			
0	144 (50.9%)	123 (58.9%)	21 (28.4%)
1	79 (27.9%)	47 (22.5%)	32 (43.2%)
2	34 (12.0%)	22 (10.5%)	12 (16.2%)
>2	26 (9.2%)	17 (8.1%)	9 (12.2%)
Previous preeclampsia	45 (15.6%)	43 (20.7%)	2 (2.5%)
Chronic hypertension	72 (26.2%)	44 (22.6%)	8 (35.0%)
Chronic kidney disease	25 (9.1%)	16 (8.2%)	9 (11.3%)
Pre‐pregnancy diabetes	14 (5.1%)	4 (2.1%)	10 (12.5%)
Booking blood pressure, mm Hg, mean (SD)			
Systolic	133 (21)	138 (20)	120 (19)
Diastolic	84 (15)	87 (14)	74 (13)
Gestation at sampling first test, median (IQR)	30^+6^ (27^+0^‐33^+5^)	31^+0^ (32^+5^‐34^+2^)	29^+3^ (26^+6^‐32^+23^)
Gestation at sampling repeat test, median (IQR)	34.0 (31^+3^‐36^+0^)	34^+0^ (32^+2^‐36^+5^)	33^+0^ (30^+1^‐35^+0^)
Gestation at delivery, median (IQR)	37^+0^ (35^+0^‐38^+2^)	37^+0^ (35^+0^‐38^+2^)	37^+0^ (35^+^^2^‐37^+5^)
Preterm delivery <37 wk, n (%)	130 (45.0%)	93 (44.5%)	37 (46.3%)
Small for gestational age (<10th centile)^19^	80 (38.5%)	56 (26.8%)	29 (36.3%)
Birthweight (g)	2464 (1835, 2929)	2550 (1835, 3120)	2445 (2018, 2770)
Preeclampsia diagnosis, n (%)	143 (55.2%)	128 (61.2%)	31 (38.8%)
Mode of delivery, n (%)			
Spontaneous	70 (29.8%)	61 (34.3%)	25 (31.3%)
Assisted	23 (9.8%)	20 (11.2%)	4 (5.0%)
Elective cesarean section	100 (42.6%)	65 (36.5%)	37 (46.3%)
Emergency cesarean section	42 (17.9%)	32 (18.0%)	14 (17.5%)

Abbreviations: IQR, interquartile range; PlGF, placental growth factor.

The median test interval between the first and second test was 15 days (interquartile range [IQR] 10‐28 days) and all values, including outliers, were included. In those testing with an initial PlGF of >100 pg/mL (133 women), the median test interval was 22 days (IQR 14‐38 days). In those testing with an initial PlGF of 12‐100 pg/mL (125 women), the median test interval was 14 days (IQR 8‐22 days). In those testing with an initial PlGF of <12 pg/mL (31 women), the median test interval was 11 days (IQR 8‐14 days). The median gestation of participants at the first test was 31^+0^ weeks (IQR 27^+1^‐33^+4^), and at the repeat test was 34^+0^ weeks (IQR 31^+3^‐36^+1^). The overall median PlGF at the first test was 53.9 pg/mL (IQR 19‐262) and the median of the repeat PlGF test was 30.6 pg/mL (IQR 12.1‐124).

At the initial test, 46.0% (n = 133) of women had a PlGF >100 pg/mL, 43.3% (n = 125) had a PlGF of 12‐100 pg/mL and 10.7% (n = 31) had a PlGF of <12 pg/mL. Overall, 25.6% of women changed PlGF category between the first and second PlGF tests. In those testing with an initial PlGF >100 pg/mL, we determined the chance of changing to a lower category at the second test, based on the level of PlGF at the initial test. In women testing with a PlGF 101‐199 pg/mL, 75% (12/16) changed category at the second test, of those testing 200‐499 pg/mL, 31% (8/26), changed category, and of those testing >500 pg/mL, 14% (3/23) changed category. In those testing initially with a PlGF 12‐100 pg/mL, those testing 12‐19 pg/mL, 41% (8/19) changed to a PlGF <12 pg/mL at the second test, whereas those testing 20‐100 pg/mL 16% (8/50) changed to a PlGF of <12 pg/mL at the second test.

**Figure 1:**
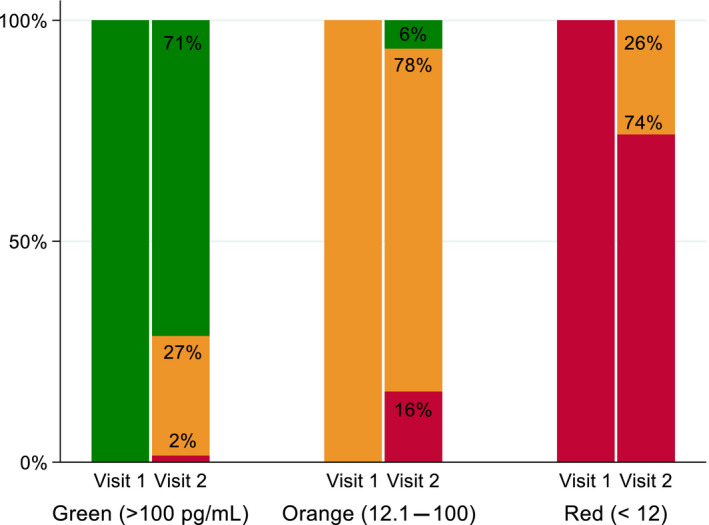
Proportion of women changing placental growth factor categories between visit 1 and visit 2

Figure 1 demonstrates the proportion of women changing PlGF categories between the first and second tests. There were no women who moved from very low PlGF (<12 pg/mL) to normal PlGF (>100 pg/mL) at the second test.

The diagnostic accuracy of PlGF (at the prespecified threshold of <100 pg/mL) in predicting preeclampsia requiring delivery within 14 days (or <37 weeks’ gestation in those recruited at 35^+0^ to 36^+6^ weeks’ gestation) for the repeat PlGF test was high: sensitivity 90.7% (95% CI 82.5‐95.9), negative predictive value 92.2% (95% CI 85.3‐96.6) and, similar to that of the first PlGF test in this cohort: sensitivity 87.5% (95% CI 67.6‐97.3), negative predictive value 97.7% (95% CI 93.5‐99.5). The test performance for the repeat PlGF test in women initially testing with a “rule out” PlGF of >100 pg/mL gave a sensitivity of 52.9% (95% CI 27.8%‐77.0%) and a negative predictive value of 91.6% (95% CI 84.1%‐96.3%) (Table 2). In a sensitivity analysis, similar high test performance was found when restricting to samples with results masked to clinicians (first test: sensitivity 94.1% (95% CI 71.3‐99.9), negative predictive value 98.5% (95% CI 91.7‐100), second test: sensitivity 95.7% (95% CI 85.5%‐99.5%), negative predictive value 95.2% (95% CI 82.8%‐99.4%) (Table 3).

**Table 2:** Test performance statistics for low placental growth factor (<100 pg/mL) in predicting preeclampsia requiring delivery within 14 d (or delivery <37 wk gestation in those presenting at 35^+0^ to 36^+6^ wk) of sample

	First PlGF test All women N = 289	Repeat PlGF test All women N = 289	Repeat PlGF test Women with initial test normal N = 133
Prevalence of clinically diagnosed preeclampsia requiring delivery (%)	8.3	29.8	12.8
Sensitivity (%)	87.5	90.7	52.9
95% CI	67.6‐97.3	82.5‐95.9	27.8‐77.0
n/N	21/24	78/86	9/17
Specificity (%)	59.1	46.8	75.0
95% CI	42.9‐55.2	39.8‐53.9	66.1‐82.6
n/N	130/265	95/203	87/116
Positive predictive value (%)	13.5	41.9	23.7
95% CI	8.5‐19.8	34.8‐49.4	11.4‐40.2
n/N	21/156	78/186	9/38
Negative predictive value (%)	97.7	92.2	91.6
95% CI	93.5‐99.5	85.3‐96.6	84.1‐96.3
n/N	130/133	95/103	87/95
Positive likelihood ratio	1.72	1.70	2.12
95% CI	1.42‐2.08	1.47‐1.97	1.22‐3.66
Negative likelihood ratio	0.25	0.20	0.63
95% CI	0.09‐0.74	0.10‐0.39	0.37‐1.05
ROC area	0.79	0.78	0.65
95% CI	0.68‐0.89	0.72‐0.84	0.49‐0.81

Abbreviations: CI, confidence interval; N, total number; PlGF, placental growth factor; ROC, receiver operating characteristic.

**Table 3:** Test performance statistics for low placental growth factor (<100 pg/mL) in predicting preeclampsia requiring delivery within 14 d (or delivery <37 wk gestation in those presenting at 35^+0^ to 36^+6^ wk) of sample in subset of women with masked placental growth factor values

	First PlGF test All women N = 158	Repeat PlGF test All women N = 158	Repeat PlGF test Women with initial test normal N = 65
Prevalence of clinically diagnosed preeclampsia requiring delivery (%)	10.8	29.7	7.7
Sensitivity (%)	94.1	95.7	60.0
95% CI	71.3‐99.9	85.5‐99.5	14.7‐94.7
n/N	16/17	45/47	3/5
Specificity (%)	45.4	36.0	66.7
95% CI	37.0‐54.0	27.1‐45.7	53.3‐78.3
n/N	64/141	40/111	40/60
Positive predictive value (%)	17.2	38.8	13.0
95% CI	10.2‐26.4	29.9‐48.3	2.8‐33.6
n/N	16/93	45/116	3/23
Negative predictive value (%)	98.5	95.2	95.2
95% CI	91.7‐100.0	83.8‐99.4	83.8‐99.4
n/N	64/65	40/42	40/42
Positive likelihood ratio	1.72	1.50	1.80
95% CI	1.42‐2.09	1.29‐1.74	0.81‐4.01
Negative likelihood ratio	0.13	0.12	0.60
95% CI	0.02‐0.88	0.03‐0.47	0.20‐1.78
ROC area	0.80	0.78	0.58
95% CI	0.70‐0.90	0.71‐0.86	0.23‐0.94

Abbreviations: CI, confidence interval; N, total number; PlGF, placental growth factor; ROC, receiver operating characteristic.

**Figure 2:**
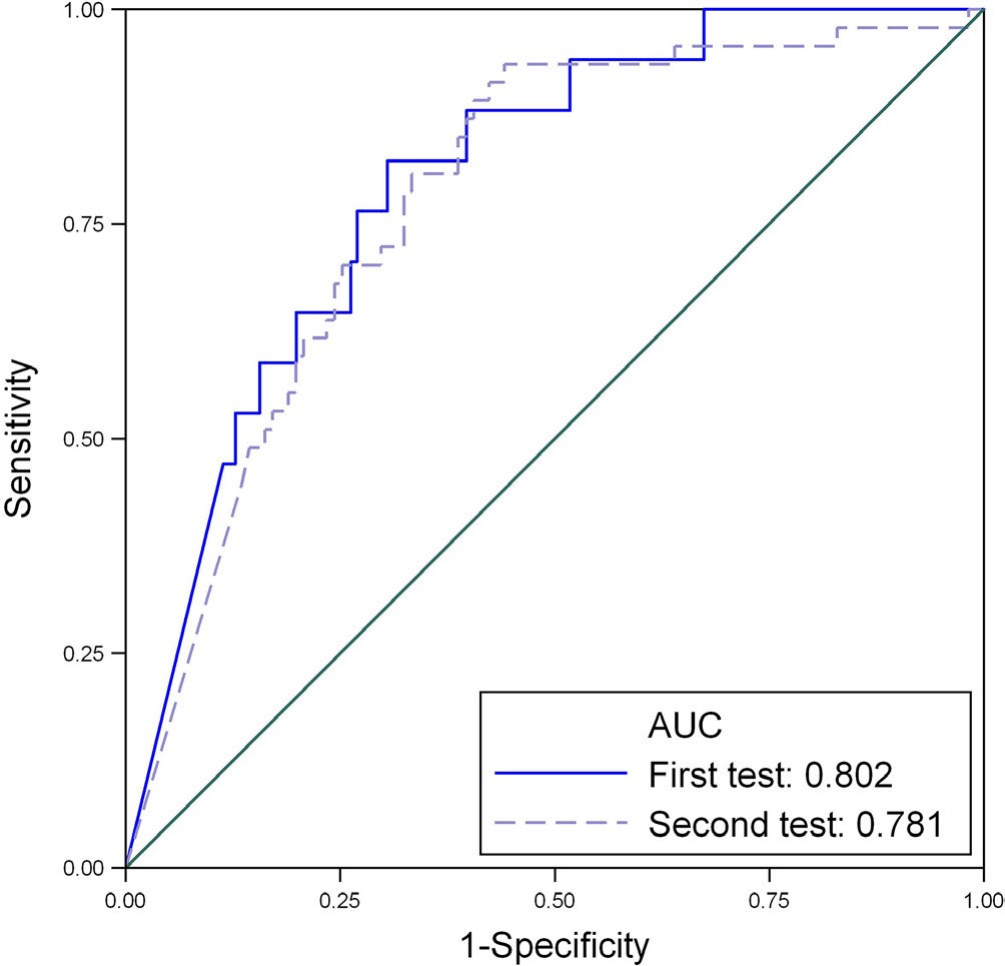
Area under receiver operating curve (AUC) for first and second test for low placental growth factor (<100 pg/mL) in predicting preeclampsia requiring delivery in 14 d from first and second tests in subset of women with masked placental growth factor values

Table 3 and Figure 2 present the diagnostic accuracy of the first and second PlGF tests in predicting preeclampsia requiring delivery within 14 days (or delivery <37 weeks in those recruited at 35^+0^ to 36^+6^ weeks’ gestation) in those undergoing masked PlGF testing (158 women). The ROC area for the repeat test is 0.78 (95% CI 0.72‐0.84), very similar to that for the first test (0.80, 95% CI 0.68‐0.89).

We investigated using the absolute difference in PlGF concentration, and the ratio of the values, and found that these measures of deterioration (eg, differences <0 and ratios <1) gave areas under the ROC curve of 0.47 and 0.53, respectively.

**Figure 3:**
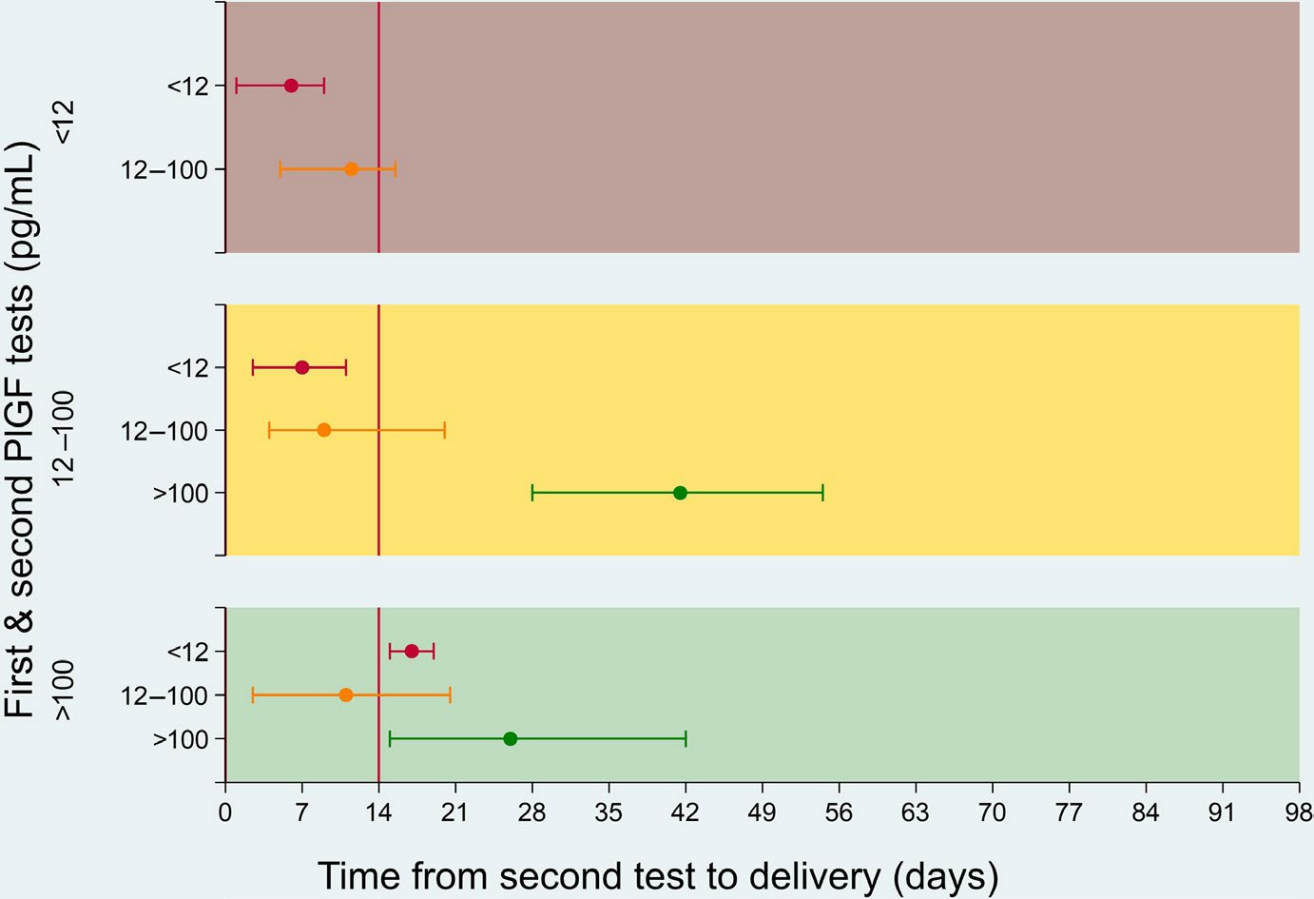
Time to delivery for preeclampsia (median, interquartile range) stratified by first placental growth factor (PlGF) concentration. Shading represents category at the first test, and line color indicates category at the second test. Red indicates very low PlGF (<12 pg/mL), orange, low PlGF (12‐100 pg/mL) and green, normal PlGF (>100 pg/mL)

Time to delivery in days by the second PlGF result, stratified by the initial PlGF sample, is given in Figure 3 (with accompanying values in Table 4). The time to delivery was similar whether the test was on repeat or initial presentation, and trends across the sub‐categories remained similar regardless of initial test category.

**Table 4:** Time to delivery for preeclampsia (median, interquartile range) stratified by first placental growth factor concentration

Category	Time to delivery (median, IQR)	Time to delivery (median, IQR)	Category	Time to delivery (median, IQR)
First test	First test	Second test	Second test stratified by first test	Second test
PlGF >100 pg/mL	23 (10‐35)	32 (14‐52)	PlGF >100 pg/mL	26 (15‐42)
			PlGF 12‐100 pg/mL	11 (3‐21)
			PlGF <12 pg/mL	17 (15‐19)
PlGF 12‐100 pg/mL	9 (4‐23)	7 (2‐16)	PlGF >100 pg/mL	42 (28‐55)
			PlGF 12‐100 pg/mL	9 (4‐20)
			PlGF <12 pg/mL	7 (3‐11)
PlGF <12 pg/mL	7 (2‐14)	6 (2‐10)	PlGF 12‐100 pg/mL	12 (5‐16)
			PlGF <12 pg/mL	6 (1‐9)

Abbreviations: IQR, interquartile range; PlGF, placental growth factor.

## 4 DISCUSSION

This case series analysis demonstrates that repeat PlGF testing in women with suspected preeclampsia before 37 weeks’ gestation maintains a high sensitivity and negative predictive value in determining which women are likely to need delivery for preeclampsia within 14 days. The prevalence of preeclampsia in this study is similar to that seen in other prospective cohort studies recruiting women with suspected preeclampsia.^11^, ^16^, ^20^ Women in all categories of the initial PlGF test change category, demonstrating that PlGF is a dynamic biomarker at the time of suspected clinical disease and there may be utility in repeated testing beyond the test performed at the initial clinical presentation. Nearly one‐third of women with an initial “normal” PlGF go on to have a subsequent “low” or “very low” PlGF at repeat, supporting a case for evaluating clinical benefit in repeating a PlGF sample to further stratify the women who remain at risk.

Time to delivery is markedly different for women in different initial PlGF categories. This is seen with both the first and second tests when considered in isolation. Importantly, when stratifying women by their initial PlGF test, time to delivery is markedly different between women who change or remain within their initial PlGF category between the first and second tests. This suggests that repeat PlGF testing may improve risk stratified management, signposting women for appropriate increased surveillance if they remain with suspected clinical disease and change to a lower PlGF category.

The strengths of this study include the use of multiple centers in the UK, encompassing a demographically diverse population. This is the largest study (to our knowledge) of repeat PlGF sampling in women presenting with ongoing suspected preeclampsia, and the only study to present diagnostic accuracy data of repeat PlGF samples for suspected preeclampsia requiring delivery within 14 days. This analysis of repeated PlGF measures was undertaken in a high‐risk population (women with suspected preeclampsia) with 30% preeclampsia prevalence. Results cannot be directly extrapolated to a lower risk population with a lower prevalence of preeclampsia, or to use of repeated measures in a screening capacity (eg, at a fixed time‐point in the third trimester).

Although other studies have investigated the use of longitudinal sampling of PlGF in pregnancy,^21^, ^22^, ^23^ these have focused on the prediction of preeclampsia risk with fixed repeat time‐point sampling.

This  study has some limitations. It is a retrospective analysis of a case series of women drawn from several different cohorts. In those changing PlGF category at the second test, our analysis is limited by small numbers. Some of the variability in PlGF levels may be a function of normal gestational fluctuations in pregnancy. In this study, PlGF results in some cases were masked and in others revealed to the clinical team. A previous cohort comparison study has suggested that clinician knowledge of PlGF leads to earlier gestation at delivery when compared with those undergoing masked testing.^15^ We therefore performed a sensitivity analysis of the test performance on the masked data only, to mitigate the risk that the outcome of preeclampsia requiring delivery within 14 days was influenced by clinician knowledge of revealed PlGF samples. This demonstrated that the sensitivity and negative predictive value remained high, albeit with slightly wider confidence intervals due to the smaller numbers included in the analysis.

Suspected preeclampsia is one of the most common indications for women to present to antenatal assessment services. We know from previous studies that PlGF outperforms all tests commonly used in antenatal triage settings for the diagnosis of preeclampsia at the initial clinical presentation.^11^ The high sensitivity and negative predictive value of PlGF testing is clinically valuable, as the low false‐negative rates are reassuring to women and clinicians planning follow‐up and care pathways. However, there is also a group of women who are not diagnosed with preeclampsia at the initial clinical presentation who go on to develop preeclampsia at a later point in pregnancy. Women with the most severe preeclampsia may not benefit from repeat sampling, as they may reach a diagnosis quickly or may need medically indicated delivery before a second test is taken. Given the heterogeneity in clinical presentations of suspected preeclampsia and the poor diagnostic accuracy of our current methods of clinical assessment, these data show that PlGF may be a useful diagnostic adjunct in women where there is ongoing diagnostic uncertainty. Our finding that the high sensitivity and negative predictive value appears to be maintained at repeat sampling, suggests that there may be benefit in repeated testing for women in whom a diagnosis remains uncertain. The relatively low positive predictive value in both initial and repeat testing may lead to over‐investigation of women, but as this is a cohort at risk of serious maternal and perinatal adverse events, this is likely to be acceptable to women and clinicians.

The results of the PARROT trial demonstrated no difference in perinatal death rates with revealed PlGF testing, in contrast to other case series in the literature.^15^ It may be that in order to prevent avoidable stillbirths, repeated PlGF testing is indicated alongside ultrasound fetal surveillance as a means of disease monitoring. Further work is required to assess the impact of repeat PlGF testing on important maternal and perinatal outcomes, to ascertain rates of clinical outcomes in those changing categories at the second test, and the cost‐effectiveness of repeat PlGF testing.

Women testing closer to the threshold values of 12 or 100 pg/mL at the initial test are more likely to change category at the second test. Further work should therefore determine whether PlGF should be considered a continuous variable, as with other markers such as platelet count or serum creatinine, and whether absolute values or gestation referenced centiles could lead to improved risk discrimination beyond the three currently utilized categories of PlGF level. A prospective study of repeat PlGF sampling could also explore the different scenarios in which repeat testing may be indicated, for example in all women to play a role in ongoing risk stratification, in those without a definitive diagnosis but whom there is an ongoing clinical suspicion of disease, or even in those with confirmed preeclampsia for prognosis of pregnancy outcome.

## 5 CONCLUSION

Suspected preeclampsia is a clinical challenge encountered daily by clinicians in maternity triage settings. The diagnostic accuracy of detection of preeclampsia requiring delivery within 14 days remains high with repeat PlGF testing. Repeat testing may be clinically useful to risk stratify women presenting suspected preeclampsia if they present with ongoing symptoms of disease.

## CONFLICT OF INTEREST

The authors have stated explicitly that there are no conflicts of interest in connection with this article.
